# ST-AFN: a spatial-temporal attention based fusion network for lane-level traffic flow prediction

**DOI:** 10.7717/peerj-cs.470

**Published:** 2021-04-22

**Authors:** Guojiang Shen, Kaifeng Yu, Meiyu Zhang, Xiangjie Kong

**Affiliations:** College of Computer Science and Technology, Zhejiang University of Technology, Hangzhou, China

**Keywords:** Attention Mechanism, Spatial-temporal network, Lane-level traffic flow prediction

## Abstract

Traffic flow prediction is the foundation of many applications in smart cities, and the granular precision of traffic flow prediction has to be enhanced with refined applications. However, most of the existing researches cannot meet these requirements. In this paper, we propose a spatial-temporal attention based fusion network (ST-AFN), for lane-level precise prediction. This seq2seq model consists of three parts, namely speed process network, spatial encoder, and temporal decoder. In order to exploit the dynamic dependencies among lanes, attention mechanism blocks are embedded in those networks. The application of deep spatial-temporal information matrix results in progresses in term of reliability. Furthermore, a specific ground lane selection method is also proposed to ST-AFN. To evaluate the proposed model, four months of real-world traffic data are collected in Xiaoshan District, Hangzhou, China. Experimental results demonstrate that ST-AFN can achieve more accurate and stable results than the benchmark models. To the best of our knowledge, this is the first time that a deep learning method has been applied to forecast traffic flow at the lane level on urban ground roads instead of expressways or elevated roads.

## Introduction

Short-term traffic flow forecasting analyzes the historical data to complete the prediction of future traffic conditions (Kong et al., 2021). It can well fulfill various requirements in a smart city (Kong et al., 2020b; Kong et al., 2020a), such as travel route guidance, congestion relief, and road planning (Do et al., 2019). With the rapid development of AI and connected vehicle technology, smart cities push for higher-precision and fine-grained requirements (Kong et al., 2020b; Kong et al., 2020a) for the traffic flow predictions.

**Figure 1:**
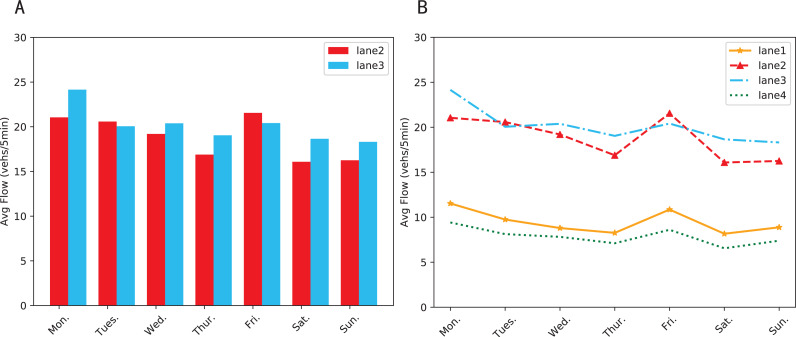
Average traffic volume of each lane during the week. (A) The differences in traffic condition between two straight lanes. (B) Differences in traffic condition between four lanes under the same intersection.

Lane-level traffic forecasting is developed from traditional prediction, which utilizes lanes parameters instead of whole road segments (Shen, Zhao & Kong, 2021). It can not only make the prediction more refined, but also lay the foundation for subsequent advanced applications such as high-precision navigation, unmanned vehicle technologies, and cooperative vehicle infrastructure system (Liao et al., 2018). Even though it is one of the key issues of Intelligent Transportation System (ITS), it is often overlooked (Liu, Zhou & Li, 2019). According to statistics, lane-level predictions only account for less than 10% of the total (Gu et al., 2019). Apart from the difficulty in obtaining lane-based data, the main reason is the mistaken assumption that different lanes have similar traffic patterns. Recently, some studies have proved the independence of lanes (Daganzo, 2002). The real-world data collected by us strongly supports this theory. As shown in Fig. 1A, lane 2 and lane 3 are the two straight lanes under the same junction and the difference in average traffic volume between them can be up to 21.34%. Figure 1B shows that the lane with the highest traffic volume (lane 3) is 2.63 times the lane with the lowest volume (lane 4). Compared to road sections or road networks, lanes are more susceptible to interference, tending to show different states when being affected by accidents, bad weather, and other factors.

Recently, several research groups have studied the lane traffic models and prediction in deep learning methods. Gu et al. (2019) applied entropy-based gray correlation to analyze the dependency between lanes by employing the network structure of Long Short-Term Memory (LSTM) and Gate Recurrent Unit. Ke et al. (2020) expanded each lane data into each channel matrix, as the input of Convolutional Neural Networks (CNN), and then obtained the final result after fusion. Xie et al. (2019) utilized Deep Believe Networks (DBN) and LSTM to build a vehicle lane change model that includes lane change decision and lane change implementation. There are still some limitations with these studies: firstly, the traffic volume on the low-speed ground roads is smaller and the speed of vehicles is relatively slower when compared with highways. Due to the complex topographic road structures and various traffic signal schemes, there exist more diversified traffic patterns (Kamal, Hayakawa & Imura, 2020). Secondly, in the forecasting, the state of a traffic node is related to its own historical situation and neighboring nodes. The processing of temporal and spatial sequence cannot be done through simple parallel analysis or linear fusion. How to deal with it still remains a thorny issue.

In this paper, we present a spatial–temporal attention mechanism based fusion network (ST-AFN) to address these issues. The contributions of this study mainly involve the following three aspects:

• ST-AFN uses the bidirectional LSTM as the basic unit, and is designed with a structure of seq2seq. Spatial attention blocks and temporal attention blocks are embedded in the encoder network and decoder network respectively. The blocks solve the problem of long-distance dependence efficiently in parallel, and capture deep level characteristics. Specially, the output matrix of the speed process network is fused with the result of spatial encoder to construct the information matrix. Continued analysis of this matrix can lead to the final volume prediction results.
• Based on the traffic volume, vehicle speeds and complex road structures, we consider the relationship between lanes in detail and adopt a novel ground road lane selection strategy.
• Real-world traffic data verifies that ST-AFN outperforms the four baseline methods (including the state of the art lane-level forecasting method FDL), in both accuracy and stability.

## Related Works

Throughout the history of traffic flow prediction, the methods can be roughly divided into three categories: parametric, non-parametric, and deep learning (Xiang, Lu & Liu, 2011; Lint & Hinsbergen, 2012). Parametric methods (e.g., Autoregressive Integrated Moving Average mode, Linear regression model) are based on the assumption that traffic runs smoothly, a deterministic model structure is established, and that the various parameters in the structure are determined by processing real data (Lippi, Bertini & Frasconi, 2013; Chan, Dillon & Singh, 2012). However, these models depend on the assumption of stationary, cannot reflect the nonlinearity and uncertainty characteristics of traffic data (Zhao et al., 2019). Non-parametric methods (e.g., Support Vector Machines, Markov model) can be more flexible due to their variable structure and parameters to cope with all kinds of unexpected situations in traffic (Hinsbergen et al., 2011; Wu, Wei & Su, 2003).

As one of the branches of machine learning, deep learning has promoted the development of various researches with its strong learning ability and excellent portability (LeYun, Bengio & Hinton, 2015; Kong et al., 2019). Wu et al. (2018) trained the traffic data in three categories: hourly, daily, and weekly with a fully connected neural network in turn, and then fused those outputs to obtain the final result. Liu et al. (2019a) and Liu et al. (2019b) utilized a deep residual network, and proposed a Traffic State Index to measure the congestion of a region. Li et al. (2018) modeled the vehicle driving as a random diffusion process on a directed graph, introduced a diffusion convolutional recurrent neural network and achieved accurate prediction results. Zhao et al. (2019) combined Gate Recurrent Unit (GRU) and Graph Convolution Network (GCN): used GRU to process time dependence and GCN to process spatial dependence, finally completed the sub-region traffic flow prediction. In addition to traffic flow forecasting, deep learning is also widely used in other areas of urban computing. Liu et al. (2019a) and Liu et al. (2019b) presented a graph processing based traffic estimation system. The system decomposed the numerous computations involved in non-linear models, and used crowd density and vehicle to predict city scale traffic patterns. Zhang, Zhang & Qi (2017) designed the residual network to model the temporal proximity and periodicity. The output of two units were aggregated, and were given different weights. Wang et al. (2017) proposed an automobile demand model. This model consisted of three residual sub-networks, which were used to analyze weather data, traffic data, and order data. Tong et al. (2017) used taxi trajectory data to forecast taxi demand. Shen et al. (2019) used Siamese CNN for multi-pedestrian tracking.

Attention mechanism was first applied to the research of natural language processing (NLP) (Bahdanau, Cho & Bengio, 2015). It emphasizes the reasonable allocation of limited computing power when facing problems (Chaudhari et al., 2019). Due to the excellent effect, this mechanism has made breakthroughs in NLP (Maharjan et al., 2018), and computer vision (CV) (Li et al., 2019). At the same time, attention mechanism is also introduced in transportation research. Guo et al. (2019) added attention blocks to GCN, and fused three subnet’s output to obtain the final prediction result. Liu et al. (2019a) and Liu et al. (2019b) expanded a variety of machine learning result vectors into a multi-channel spatio-temporal matrix and used attention when calculating the weights between channel. Hao, Lee & Zhao (2019) used the attention mechanism twice in the encoder and decoder, and used the embedding layer to combine external factors such as weather and emergencies. Zhang et al. (2019) used GCN to extract spatial features, then inputted the features into the seq2seq model, the attention blocks were embedded in the encoder.

### Problem statement

Before ST-AFN is introduced in detail, this section will describe the specific ground road lane selection strategy and traffic flow prediction problem.

**Figure 2:**
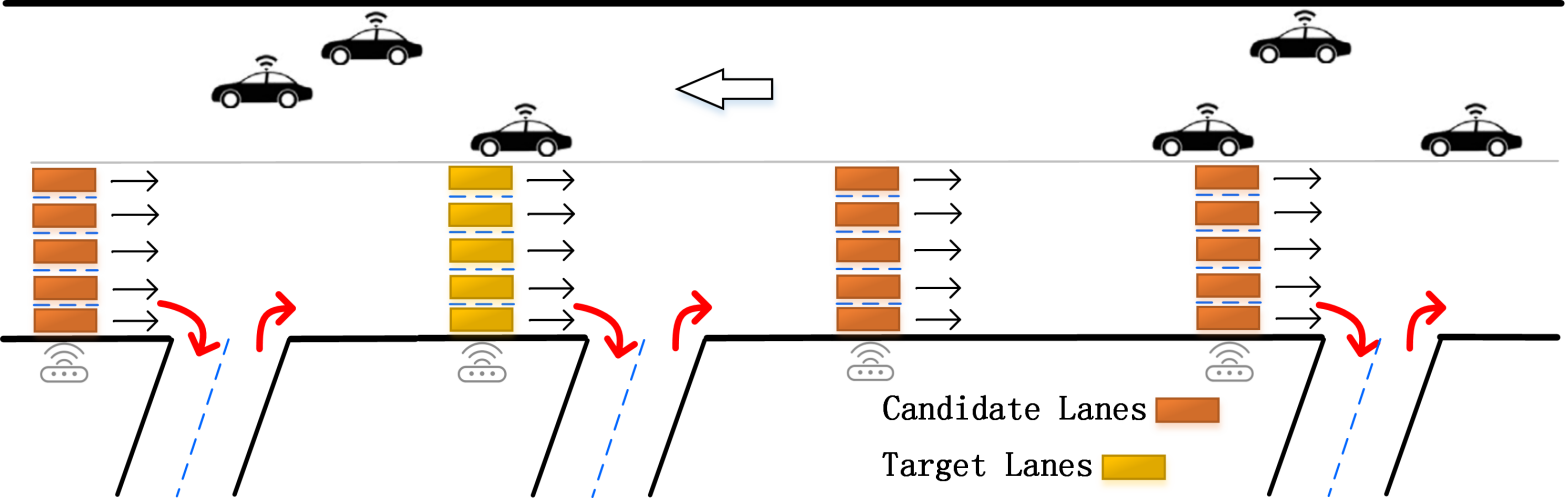
Lanes selection in previous studies.

**Definition 1: Lane Selection.** In the previous lane-level traffic flow prediction research, the experimental data came from high-ways or elevated freeways. Under this road condition, following is the corresponding solution: when on high-ways or elevated freeways, the long and straight roads are divided into multiple sub-sections based on the sensors in the main line and the ramp as shown in Fig. 2. There are often some shortcomings in the corresponding strategies under this research background: firstly, the width of the ramp is narrow, which easily becomes a bottleneck in the morning and evening peaks with dense traffic. Existing strategy does not consider a large number of inflow and outflow vehicles in the ramp (as shown by the red arrows in Fig. 2) which may lead to inaccurate results. Secondly, due to the limitation of the research background, the experiment can only use a single straight flow lane as the research object. It has poor scalability and limited practical application range. In comparison, urban ground roads have the characteristics of a higher proportion of total road mileage, wider coverage area, and more complex traffic patterns (Kamal, Hayakawa & Imura, 2020). However, there is still no mature and efficient lane selection strategy for ground roads.

**Figure 3:**
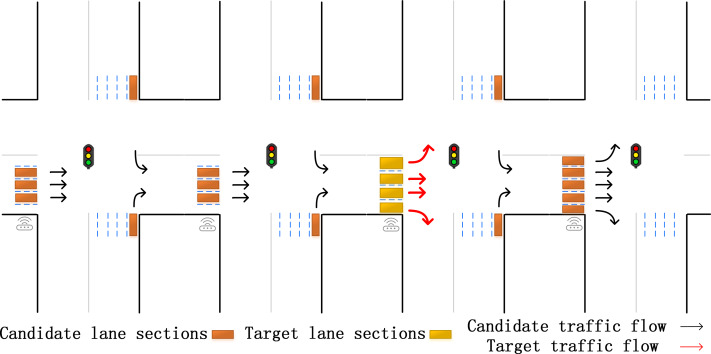
Our specific lanes selection. The candidate lanes participate in the training of the neural network, and the target lanes are used as the training targets.

Considering the above research strategies and the characteristics of the ground road network, this paper proposes the selection strategy shown in Fig. 3. On the ground roads in urban areas, the intersections are highly correlated. The basis for selecting the lanes with intersections alone is sufficient to support the subsequent prediction. This method not only takes the adjacent lanes below the same intersection into consideration, but also selects lanes in the upstream and downstream intersections, including straight, and often overlooked left-turn and right-turn lanes. This strategy is based on the physical connection structure of the road, and perfectly captures the information in lanes. In this way, it not only can improve the prediction accuracy, but also broaden the application range.

**Definition 2:** Flow Prediction. After the lane traffic data is collected, we divide the training data set into *n* parts at fixed time intervals. For convenience, this article records the total number of lanes under *k* intersections as *m*. Each lane has *h*- dimensional attributes. The traffic characteristic parameters can be denoted by an *n* × *h* × *m* matrix *T*^±^. In detail ${T}_{q}^{+}$ denotes the forward volume, and ${T}_{s}^{+}$ denotes forward speed. The predicted goal can be described as:

(1) \begin{eqnarray*} \left[ {X}_{n+1}^{q+},{X}_{n+2}^{q+},\ldots ,{X}_{n+\tau }^{q+} \right] =\mathrm{f} \left( \mathrm{g} \left( {T}_{q}^{+} \right) \oplus \mathrm{p} \left( {T}_{s}^{+} \right) \right) \end{eqnarray*}

where g is the encoding processing function, p is the speed processing function, f is the final decoding and output function, *X*^*q*+^ is the lane volume value, *τ* is the forecast time length.

### ST-AFN framework

**Figure 4:**
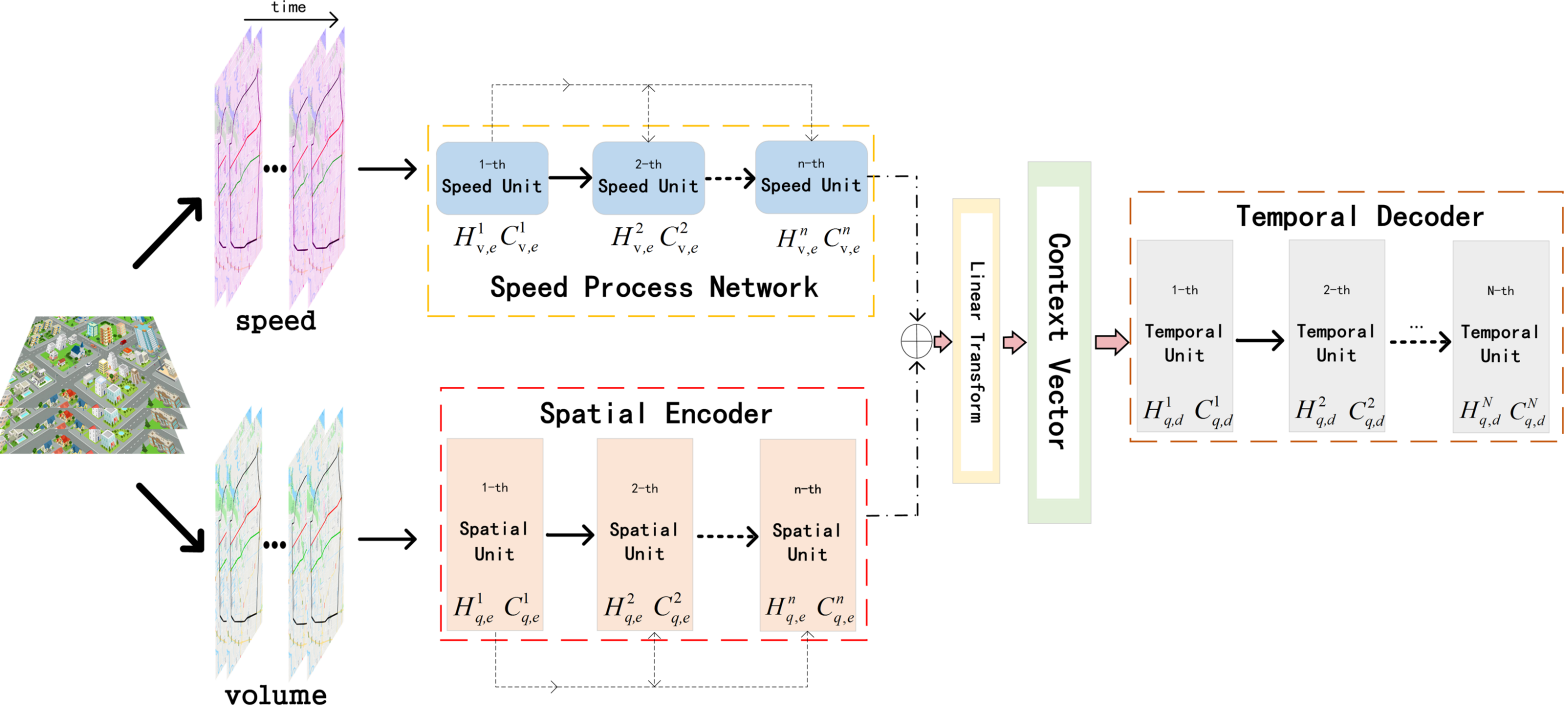
The structure of ST-AFN.

ST-AFN is mainly composed of three parts: deep speed processing network, the spatial encoder, and the temporal decoder. Deep bidirectional LSTM are connected in series to form the speed processing network. The encoder with the spatial attention blocks are utilized to analyze the spatial characteristics of the traffic parameters. Then the output of the above two networks are merged to build the information matrix which is the input of decoder. After the decoder extracting temporal feature, we use full connected layer (FC) to complete the final prediction, as shown in Fig. 4. The following will introduce each network in order.

### Speed process network

The current traffic situation of the road section is closely related to its upstream and downstream sections. The upstream traffic flows toward the downstream section, and the state of the downstream section (such as congestion) will gradually accumulate, which in turn will impact the upstream traffic flow. In the direction of the time dimension, bidirectional LSTM is superimposed and fused by forward LSTM and backward LSTM. While overcoming the problems of gradient disappearance and gradient explosion, it also considers the forward and backward propagation of sequential data.

**Figure 5:**
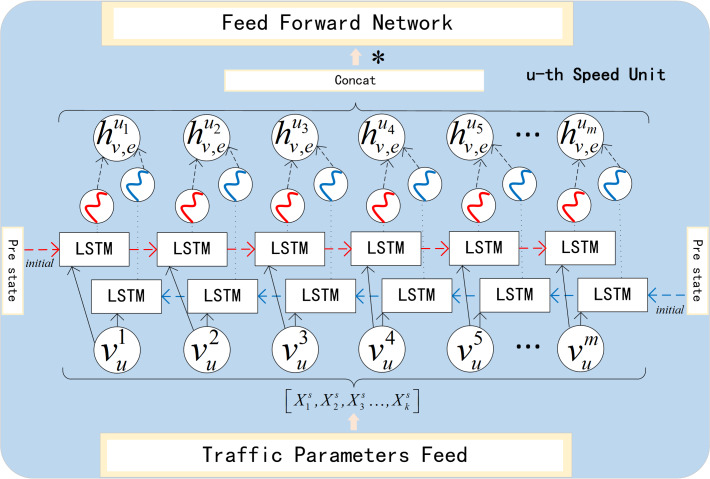
Speed process unit.

As shown in the Fig. 5, ${h^{\leftarrow }}_{ve}^{{u}_{l}}$ is derived from the forward LSTM, and ${h^{\rightarrow }}_{ve}^{{u}_{l}}$ is derived from backward LSTM. They represent the forward hidden state and backward hidden state in *u*-th unit. Correspondingly, ${c^{\rightarrow }}_{ve}^{{u}_{l}}$ and ${c^{\leftarrow }}_{ve}^{{u}_{l}}$ represent the forward cell state and backward cell state respectively. After each subunit completes forward and backward propagation, ${h^{\rightarrow }}_{ve}^{{u}_{l}}$ and ${h^{\leftarrow }}_{ve}^{{u}_{l}}$ are concatenated to form ${h}_{ve}^{{u}_{l}}$.

(2) \begin{eqnarray*}{H}_{ve}^{u}={W}_{ve}^{u}\cdot [{h}_{ve}^{{u}_{1}},{h}_{ve}^{{u}_{2}},\ldots ,{h}_{ve}^{{u}_{m}}]^{\mathrm{T}}+{b}_{ve}^{u}\end{eqnarray*}

where ${h}_{ve}^{u}$ is the result of splicing ${h}_{ve}^{{u}_{l}}$ (*l* = 1, 2, …, *m*), and then it is changed linearly to get the final hidden state ${H}_{ve}^{u}$.${W}_{ve}^{u}$ is the weight matrix and ${b}_{ve}^{u}$ is the bias term. ${C}_{ve}^{u}$ is calculated at the same time, representing the final cell state. Then ${C}_{ve}^{u}$ and ${H}_{ve}^{u}$ are used for initialization in the next unit, and are collected to build the context vector.

### The spatial encoder

**Figure 6:**
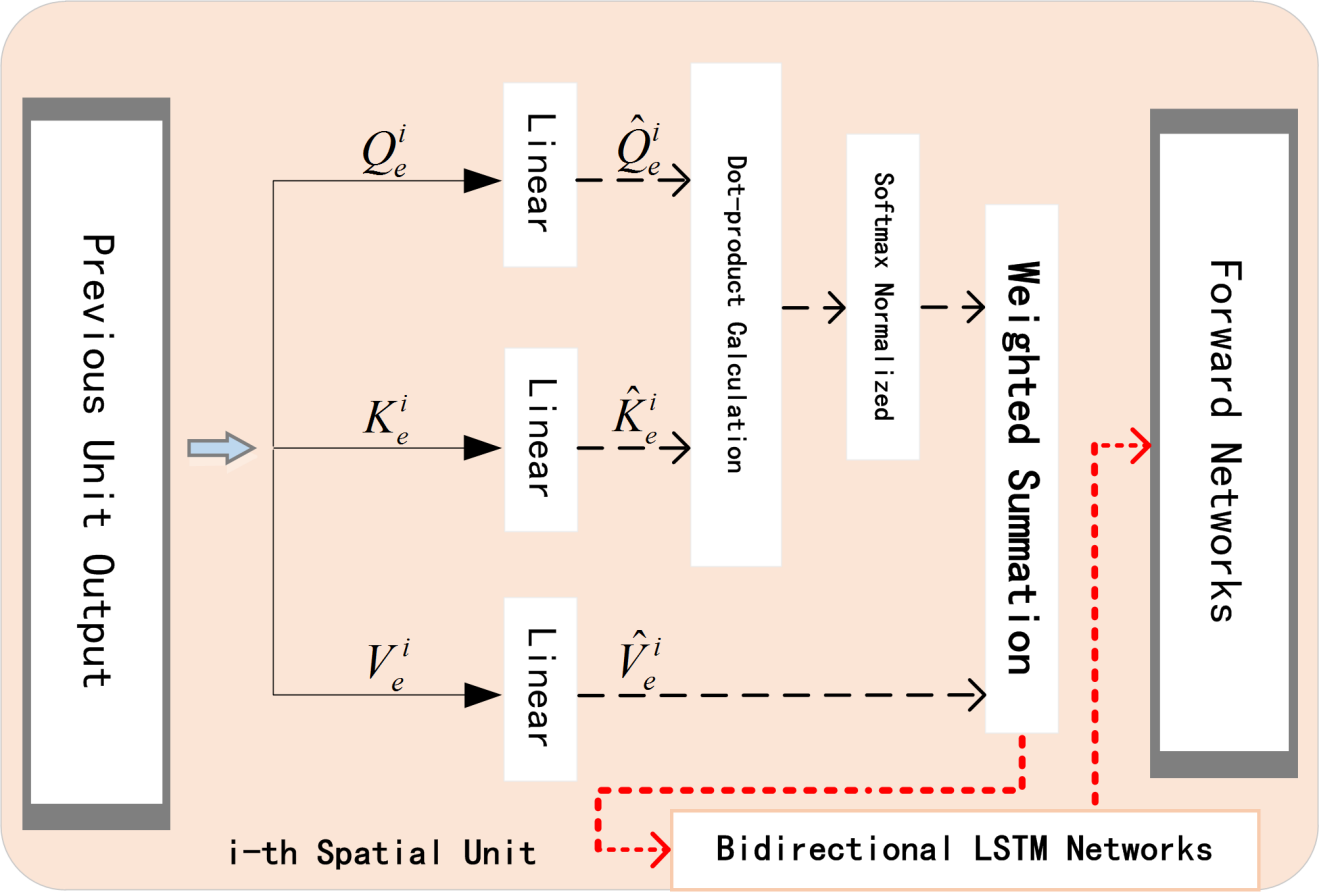
Spatial encoder unit.

When predicting traffic flow, the processing methods of time dependence and space dependence directly affect the accuracy of the experimental results. The application of attention mechanism can help networks to accurately analyze the dependencies between lanes at each moment in real time. After selecting the target lane, it pays more attention to lanes with high correlation, reduces the weight value of irrelevant lanes, and efficiently allocates weights dynamically and optimally in a parallel strategy. The proposed encoder network is shown in the Fig. 6.

The encoder with embedded spatial attention mechanism blocks is composed of another bidirectional LSTM network. The spatial blocks in the unit can be summarized as: firstly we transform query ${Q}_{e}^{i}$, key ${K}_{e}^{i}$, and value ${V}_{e}^{i}$to get ${\hat {Q}}_{e}^{i}$, ${\hat {K}}_{e}^{i}$, and ${\hat {V}}_{e}^{i}$. Secondly, we calculate the dot product and use Softmax function to normalize it. Finally, we get the weights. The attention mechanism formula in *i*-th spatial unit is:

(3) \begin{eqnarray*}{e}_{i}^{j}=\tanh \nolimits \left( {W}_{ae}^{i}\cdot \left[ {H}_{qe}^{i-1},{C}_{qe}^{i-1},{X}_{i}^{{q}_{j}^{+}} \right] +{b}_{ae}^{i} \right) \end{eqnarray*}

where ${H}_{qe}^{i-1}$ is the final hidden state of *i* − 1- th spatial unit, ${C}_{qe}^{i-1}$ is the final cell state. ${X}_{i}^{{q}_{j}}$ represents the volume of *j*- th lane in current unit. ${W}_{ae}^{i}$ and ${b}_{ae}^{i}$ respectively represent the weight item and bias item, tanh is one of the activation functions.

Now, we have obtained ${e}_{i}^{j}$ for each lane. Then a softmax function is used for the normalization, and get attention weight ${\alpha }_{i}^{j}$ for each candidate lane. Finally we weight the original data ${X}_{i}^{{q}_{j}^{+}}$ with ${\alpha }_{i}^{j}$ to get ${\hat {X}}_{i}^{{q}^{+}}$, which will be the input data of *i*- th Spatial Unit.

### The temporal decoder

**Figure 7:**
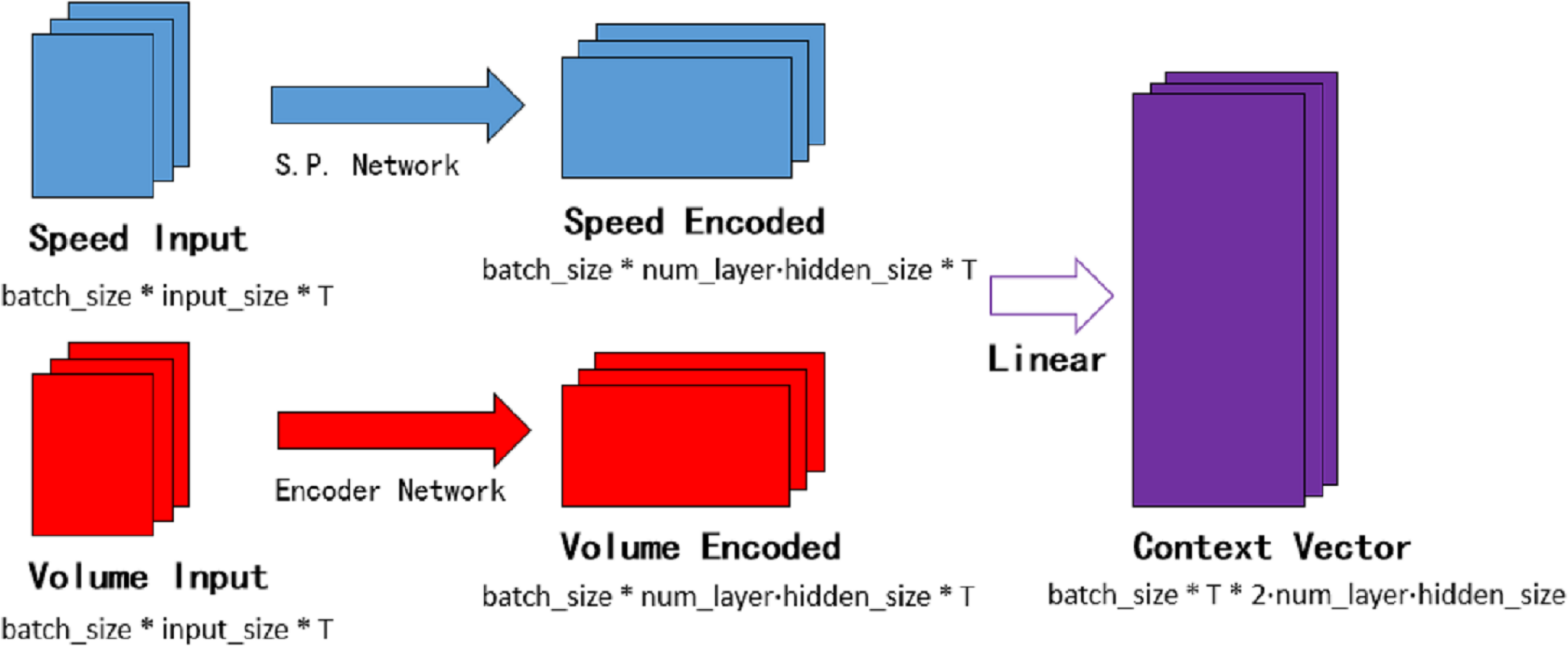
The context vector.

The temporal attention mechanism blocks are embedded in the decoder network, they are employed to distinguish the importance of each period and assign the temporal weights. Context vector *θ* is the input of the decoder network. As shown in Fig. 7, speed encoded consists of hidden layers of each subunit in the speed processing neural network. Similarly, volume encoded consists of hidden layers of each subunit in the encoder neural network. The batch_size in the figure means the number of batches per training, num_layer is the number of network loop iterations, T is the time step required for prediction, and input_size represents the number of lanes.

\begin{eqnarray*}{H}_{qe}= \left[ {H}_{qe}^{1},{H}_{qe}^{2},\ldots ,{H}_{qe}^{n} \right] \end{eqnarray*}

(4) \begin{eqnarray*}{H}_{ve}= \left[ {H}_{ve}^{1},{H}_{ve}^{2},\ldots ,{H}_{ve}^{n} \right] \theta ={W}_{qv}\cdot \left[ {H}_{ve},{H}_{qe} \right] +{b}_{qv}\end{eqnarray*}

where ${H}_{qe}^{n}$ and ${H}_{ve}^{n}$ is the final hidden state in each unit of speed process network and the encoder network respectively. *H*_*ve*_ and *H*_*qe*_ are the matrices formed by the final hidden state. *W*_*qv*_ is the weight item and *b*_*qv*_ is the bias item in the linear transform.

**Figure 8:**
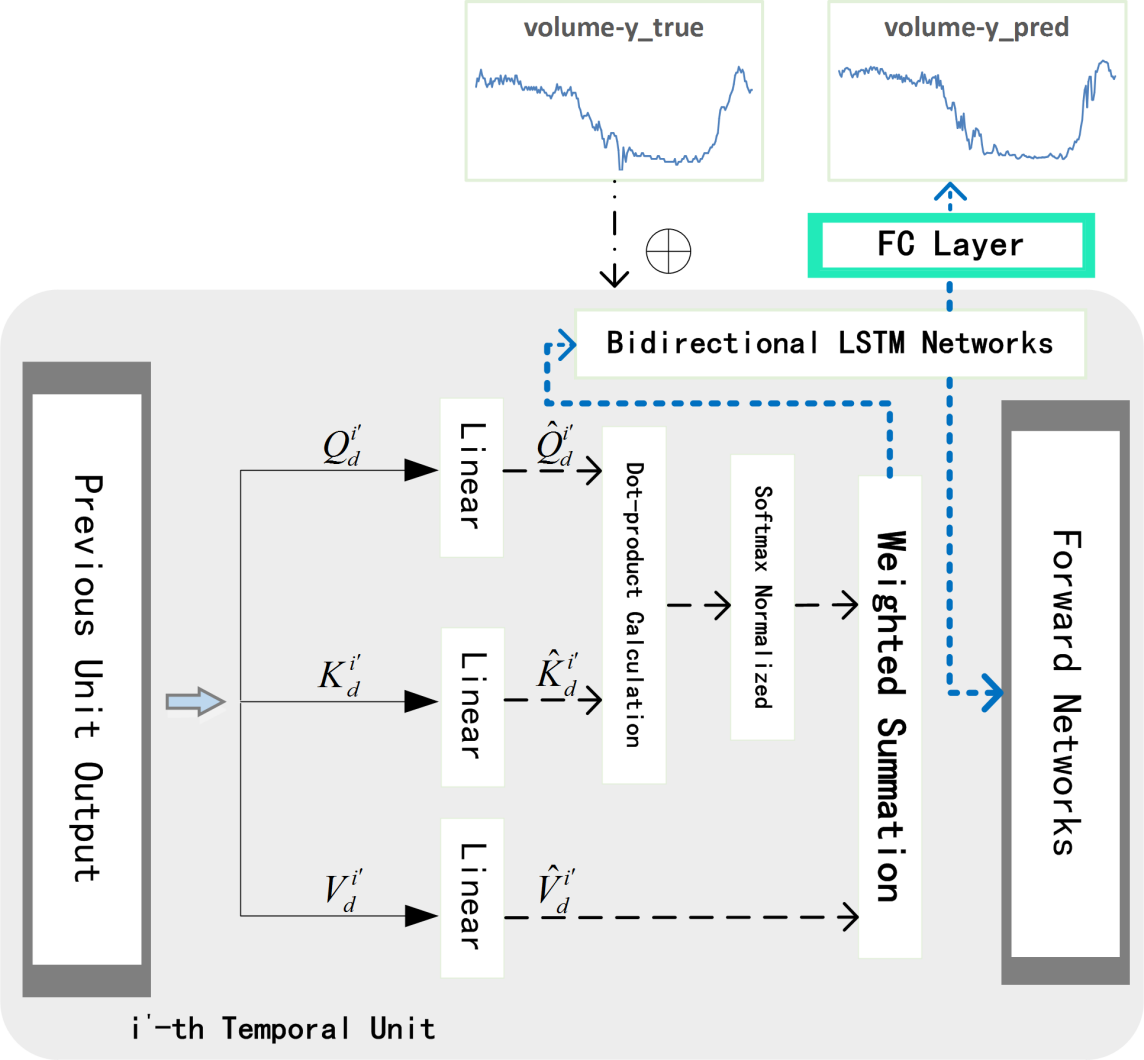
Temporal decoder unit.

As shown in the Fig. 8, the input of the *i*′- th temporal unit is *θ*_*i*′_. We mark the final hidden state and final cell state as ${H}_{qd}^{{i}^{{^{\prime}}}-1}$ and ${C}_{qd}^{{i}^{{^{\prime}}}-1}$ respectively. Temporal attention mechanism formula can be written as:

(5) \begin{eqnarray*}{d}_{{i}^{{^{\prime}}}}^{k}=\tanh \nolimits \left( {W}_{ad}^{{i}^{{^{\prime}}}}\cdot \left[ {H}_{qd}^{{i}^{{^{\prime}}}-1},{C}_{qd}^{{i}^{{^{\prime}}}-1},{\theta }_{{i}^{{^{\prime}}}}^{k} \right] +{b}_{ad}^{{i}^{{^{\prime}}}} \right) \end{eqnarray*}

where ${W}_{ad}^{{i}^{{^{\prime}}}}$ is the weight matrix and ${b}_{ad}^{{i}^{{^{\prime}}}}$ is the bias item, which are all the learnable parameters.

After obtaining ${d}_{{i}^{{^{\prime}}}}^{k}$ for each time, we apply Softmax function to normalize it, and get attention weight ${\beta }_{{i}^{{^{\prime}}}}^{k}$. Finally we weight the original data *θ*_*i*′_ with ${\beta }_{{i}^{{^{\prime}}}}^{k}$ to get ${\hat {\theta }}_{{i}^{{^{\prime}}}}$. Then ${\hat {\theta }}_{{i}^{{^{\prime}}}}$ is used together with historical real traffic flow of target lane ${y}_{his}^{{i}^{{^{\prime}}}}$ to get ${\hat {y}}_{his}^{{i}^{{^{\prime}}}}$:

(6) \begin{eqnarray*}{\hat {y}}_{his}^{{i}^{{^{\prime}}}}={W}_{f,d}^{{i}^{{^{\prime}}}} \left[ \begin{array}{@{}l@{}} \displaystyle {\hat {\theta }}_{{i}^{{^{\prime}}}}\\ \displaystyle {y}_{his}^{t} \end{array} \right] +{b}_{f,d}^{{i}^{{^{\prime}}}}\end{eqnarray*}

According to the above method, each unit in decoder network iteratively allocates temporal weights until the *N*- th unit. *N* is the size of the dimension of *θ*(excluding the temporal dimension). Finally, we concatenate ${H}_{qd}^{N}$ and ${\hat {\theta }}_{N}$, and feed the matrix into fully connected (FC) layer to get the result of predicted traffic volume ${q}_{tag}^{{i}^{{^{\prime}}}+\tau }$:

(7) \begin{eqnarray*}{q}_{tag}^{{i}^{{^{\prime}}}+\tau }={W}_{fd}^{N} \left[ \begin{array}{@{}l@{}} \displaystyle {\hat {\theta }}_{N}\\ \displaystyle {H}_{qd}^{N} \end{array} \right] +{\hat {b}}_{fd}^{N}\end{eqnarray*}

### The training process

In the training process of ST-AFN, training data are normalized and scrubbed to make up each batch, and the learning rate is adjusted using ReduceLROnPlateau: the maximum tolerance threshold is set, and the learning rate is dynamically adjusted downward using the loss value in each epoch as an index. The specific process is as follows:

**Code 1**. The training method of ST-AFN


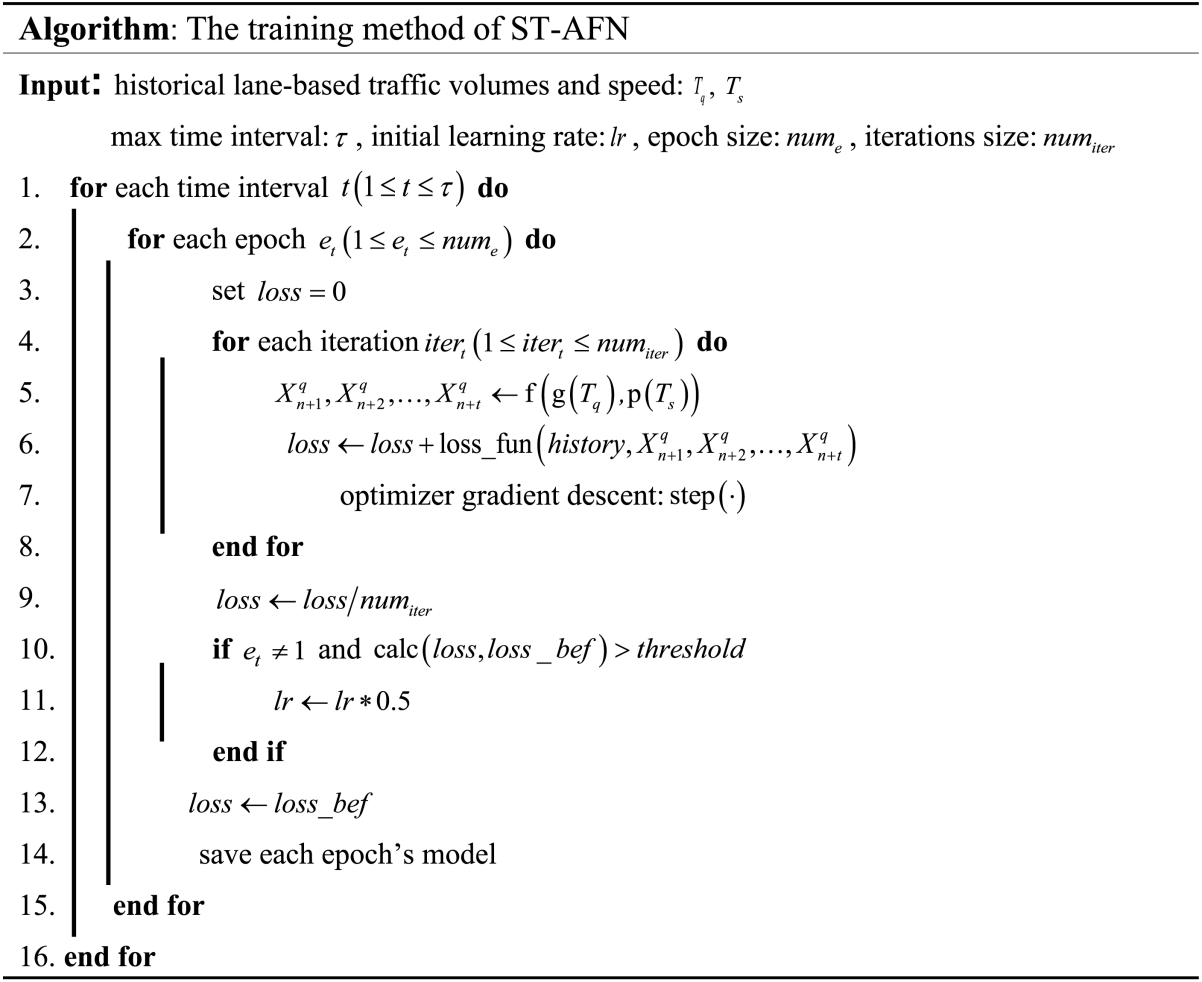


### Evaluation

In this chapter, we use the real-world traffic data to evaluate the proposed ST-AFN and the benchmark algorithms. The experiments run on 64G Ubuntu 18.04 system, which is equipped with Intel Xeon Silver and NVDIA Quadro M4000. The hyperparameters in each experimental group were set uniformly: the learning rate is set to 0.001, batch size is set to 128, the epoch size is set to 100.

### Data set

The real traffic data required for the experiment are collected in Xiaoshan District, Hangzhou, China from July 1, 2017 to October 1, 2017, for a total of 123 days. These data are collected by surveillance cameras at various intersections. The main format of the original data is shown in Table 1 (At a 4-lane intersection, lane 1 usually refers to the left-turn or U-turn lane, and lane 4 is the right-turn lane. Similarly, at a 5-lane intersection, lane 5 is the right turn lane).

Because the original data are relatively scattered, we filter out the required vehicles based on the location attributes of the vehicle’s departure time and the camera location, and eliminate the error data (the departure time is earlier than the entry time, the license plate number is empty, etc.). The distance is a fixed value, and the vehicle travel time can be obtained from the entry and departure time. What’s more, the average speed is calculated by dividing the distance between intersections by the driving time. Then we filter out the data of each lane in each intersection.

**Figure 9:**
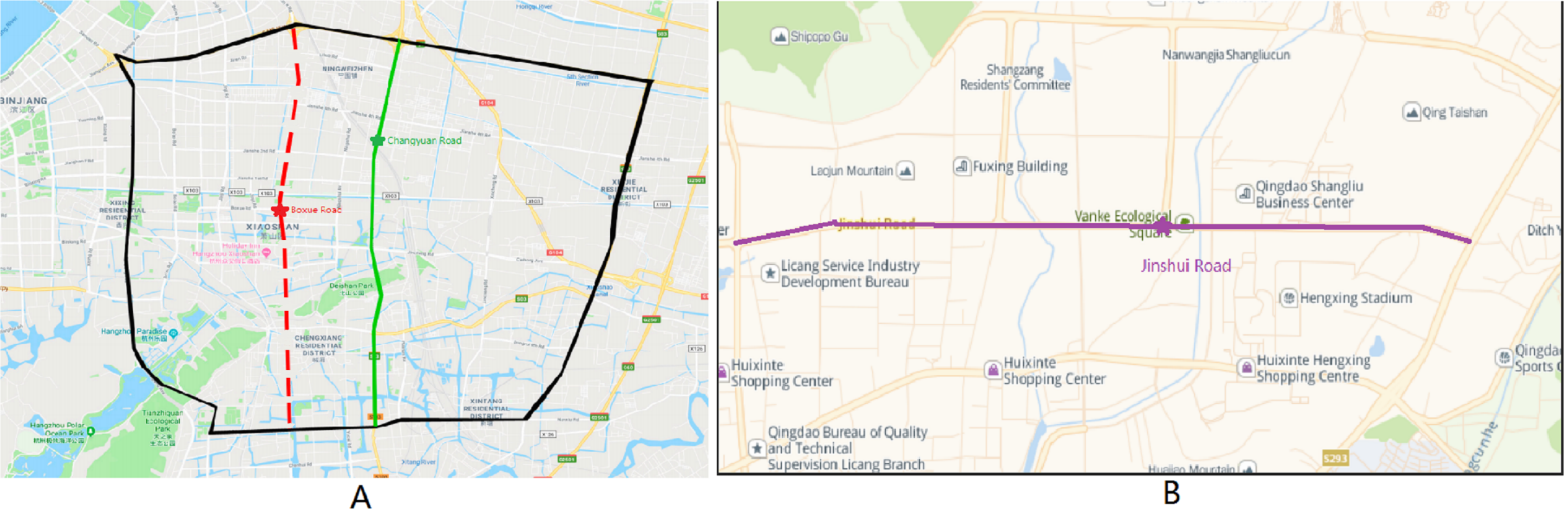
Location of experimental data. (A) Two experimental sections in Xiaoshan District, Hangzhou. (B) An experimental section in Licang District, Qingdao.

As shown in Fig. 9A, each lane under the intersection of Tonghui Road-Changyuan Road and the intersection of Shixin Road-Boxue Road is used as the target lane in the two experiments, and the remaining lanes under the road participated in the experiment as candidate lanes. Data is counted at five-minute intervals (sum the flow in five minutes and average the speed), so each detector can generate 288 records per day. Then we draw a curve according to the obtained characteristics parameters to judge the noise points and make it as smooth as possible. A large amount of data is sufficient to support the training and testing of the model. In order to reduce the interference caused by noisy data, abnormal points are repaired by adjacent normal records. Then the StandardScaler method is applied to the repaired data.

**Table 1:** The format of the original data.

**Column name**	**Annotate**
vehicleID	License plate
entryTime	Entry time of the vehicle
leaveTime	Leave time of the vehicle
vehicleType	Vehicle type
cameraID	The id of camera
cameraPos	Place where the camera is placed
laneID	Lane number the vehicle travels on
turnID	Steering of the vehicle

The data of Xiaoshan District, Hangzhou is used as the main experimental data and to prove ST-AFN’s portability, we collect real traffic data from Qingdao for verification. The data is provided by the traffic big data competition held in Qingdao in October 2019. The original data is mainly composed of laneID, speed, cameraPos, timestamp, etc., from which we selected an arterial with different directions and adjacencies as the data set. The processing strategy is similar to the above, data is counted at five-minute intervals.

As shown in Fig. 9B, each lane under the intersection of Jinshui Road-Dongchuan Road is used as the target lane.

The lanes selected for each of the three experimental datasets are presented in Table 2, arranged from top to bottom in the order in which they connect each road in a north-south direction. And in the Jinshui Road experiment set, they are arranged form left to right. The intersections where the target lanes are located in each experiment are marked by ‘*’ (e.g., *Changyuan Road-N), and the N, E, and W after the intersections are directional characters (e.g., -N for the North intersection, -N&E&W for the North, East, and West intersections).

**Table 2:** The list of selected lanes.

**Experiment sets**	**Name of lanes**	**Number of lanes**	**Lane flow**
	Zhenning Road-N&E&W	5	Left, Straight, Right
	Jianshe4 Road-N&E&W	5	Left, Straight, Right
Tonghui Road	*Changyuan Road-N	4	Left, Straight, Right
	Jianshe2 Road-N&E&W	4	Left, Straight, Right
	Jianshe1 Road-N	2	Straight
	Jianshe2 Road-N&E&W	4	Left, Straight, Right
	Jianshe1 Road-N&E&W	4	Left, Straight, Right
Shixin Road	*Boxue Road-N	4	Left, Straight, Right
	Jincheng Road-N&E&W	5	Left, Straight, Right
	Shanyin Road-N	3	Straight
	Tongchuan Road-N&E&W	5	Left, Straight, Right
	Hanchuan Road-N&E&W	3	Left, Straight, Right
Jinshui Road	Dongchuan Road-N	5	Left, Straight, Right
	Xichuan Road-N&E&W	3	Left, Straight, Right
	Longchuan Road-N	2	Straight

### Baseline methods

To compare with our model, serval parametric, non-parametric, and neural network models were selected as baseline methods (including a state of the art lane-level forecasting method).

• **SVR**: Support vector regression is a common method in traffic flow prediction. As an important branch of support vector machines, their difference is mainly in the evaluation index. The optimal hyperplane in SVM allows multiple categories to be the farthest apart, while the optimal hyperplane for SVR is the one that minimizes the sum of deviations of all sample points
• **ARIMA**: Autoregressive integrated moving average is one of the time series forecasting methods. It contains an auto regressive block and a moving average block.
• **LSTM**: LSTM composed of input gates, output gates, and forget gates is an improved version of Recurrent Neural Network (RNN).
• **TM-CNN:** TM-CNN (Ke et al., 2020) refers a two-stream multi-channel convolutional neural network. Firstly the authors convert traffic speed data and volume data into matrices. Then they expand each lane data into each channel matrix, as the input of CNN. And obtain the final result after fusion.
• **FDL:** FDL (Gu et al., 2019) refers a fusion deep learning model. Firstly, entropy-based gray relational analysis method is utilized to judge the dependency between lanes. Then they utilize the LSTM-GRU structure network to complete the lane-level speed forecast. It is one of state of the art models in lane level prediction.

### Performance metric

In this study, we evaluate the prediction results with Mean Absolute Error (MAE), Root Mean Square Error (RMSE), and Mean Absolute Percentage Error (MAPE).

(8) \begin{eqnarray*}MAE= \frac{1}{N} \sum _{i=1}^{N} \left\vert {\hat {y}}_{i}-{y}_{i} \right\vert \end{eqnarray*}

(9) \begin{eqnarray*}RMSE=\sqrt{ \frac{1}{N} \sum _{i=1}^{N}{ \left( {\hat {y}}_{i}-{y}_{i} \right) }^{2}}\end{eqnarray*}

(10) \begin{eqnarray*}MAPE= \frac{100\text{%}}{N} \sum _{i=1}^{N} \left\vert \frac{{\hat {y}}_{i}-{y}_{i}}{{y}_{i}} \right\vert \end{eqnarray*}

where *N* is the number of forecast targets, *y*_*i*_ is the history value and ${\hat {y}}_{i}$ is the forecast result.

## Result

Table 3 shows the experimental results. As can be seen, ST-AFN achieves the lowest RMSE (mean 2.541), MAE (mean 1.379), and MAPE (mean 9.720) which are much lower than baseline methods. The traditional machine learning method SVR cannot cope with this problem well, with average RMSE, average MAE, and average MAPE of 5.073, 3.913, and 14.482, respectively. The bad performance of time series method ARIMA indicates that the spatial dependency is as important as temporal dependency during the predictions and simple model structures cannot handle the complex pattern recognition between lanes. The single network LSTM perform better than ARIMA with average RMSE, average MAE, and average MAPE of 4.273, 2.866, and 13.368, respectively. The ST-AFN* (without speed-processing neural network) does not exceed FDL, which justifies the need for fusion of speed characteristic parameters and traffic flow characteristic parameters. The state of the art method, FDL, achieves accurate predictions with average RMSE, average MAE, and average MAPE of 2.772, 1.633, and 13.333, respectively.

The difference in traffic flow between weekdays and weekends is obvious, for example the peak trend is obvious in the morning and evening on weekdays, while the traffic flow is large throughout the day on weekends. We divide the data set into a training set and a test set at a ratio of 7:3. The test set has a total of 37 days, including 27 weekdays and 10 weekends. Table 4 shows the ST-AFN’s performances. It can be obtained from the results. Although the difference is small, ST-AFN has achieved better prediction results on weekdays when the morning and evening peaks are obvious.

We use the data set from Jinshui Road, Qingdao to prove ST-AFN’s portability. The data is provided by the big data competition held in Qingdao in October 2019. In this data set, compared with the state of the art lane-level forecasting method FDL, ST-AFN achieves the same better results as before. This proves the great portability of ST-AFN. There are three main reasons in our opinion. Firstly, we have developed a processing strategy for traffic data in a standard format. Secondly, the specific lane selection strategy is based on the traffic volume, vehicle speed and complex road structures. Last, ST-AFN has a strong self-learning ability and can achieve a high accuracy rate after training with temporal series data and spatial series data. The performance of ST-AFN under sparse data conditions remains to be discussed. As shown in Table 5.

**Table 3:** Comparison among different models.

**Algorithm**	**Error Index**	Tonghui/Shixin-L1	Tonghui/Shixin-L2	Tonghui/Shixin-L3	Tonghui/Shixin-L4
	MAE	4.064	3.733	3.687	4.168
SVR	RMSE	4.807	5.09	5.26	5.136
	MAPE	14.847	13.926	14.367	14.798
	MAE	3.845	3.793	3.856	3.488
ARIMA	RMSE	4.821	4.955	5.178	4.899
	MAPE	13.845	14.718	14.296	14.323
	MAE	3.054	2.987	2.576	2.845
LSTM	RMSE	3.746	4.547	4.824	3.977
	MAPE	12.798	12.854	13.452	14.369
	MAE	2.265	2.077	1.732	2.184
ST-AFN*	RMSE	3.456	3.848	3.125	3.282
	MAPE	13.063	11.985	12.457	12.783
	MAE	1.665	1.548	1.618	1.826
TM-CNN	RMSE	3.635	3.320	3.159	3.516
	MAPE	13.153	12.018	11.784	12.438
	MAE	1.793	1.615	1.488	1.634
FDL	RMSE	2.715	2.571	2.943	2.858
	MAPE	11.578	11.454	10.636	11.662
	MAE	**1.484**	**1.346**	**1.228**	**1.465**
ST-AFN	RMSE	**2.533**	**2.422**	**2.701**	**2.517**
	MAPE	**10.136**	**9.415**	**9.351**	**9.976**

**Table 4:** Comparison of the results in weekdays and weekends.

Lane ID	Weekdays				Weekends		
	MAE	RMSE	MAPE		MAE	RMSE	MAPE
Tonghui/Shixin-L1	1.473	2.527	10.114		1.506	2.596	10.328
Tonghui/Shixin-L2	1.291	2.375	9.435		1.416	2.630	9.256
Tonghui/Shixin-L3	1.214	2.617	9.304		1.311	2.820	9.483
Tonghui/Shixin-L4	1.494	2.413	10.038		1.402	2.314	9.866

**Table 5:** Comparison of the results in Jinshui Road.

**Algorithm**	**Error Index**	Jinshui-L1	Jinshui-L2	Jinshui-L3	Jinshui-L4	Jinshui-L5
	MAE	2.085	2.430	1.817	2.522	2.211
TM-CNN	RMSE	5.241 21	4.043	4.253 \2	4.350	4.520
	MAPE	12.436	12.133	11.763	12. 158	12.371
	MAE	1.812	1.745	1.684	1.783	1.812
FDL	RMSE	4.832	3.840	3.675	4.603	4.563
	MAPE	12.732	11.989	11.816	12.564	11.866
	MAE	**1.552**	**1.420**	**1.394**	**1.432**	**1.534**
ST-AFN	RMSE	**3.425**	**3.156**	**2.935**	**3.526**	**4.106**
	MAPE	**11.953**	**10.843**	**10.849**	**11.616**	**11.340**

In the field of natural language processing, the seq2seq architecture is usually used in real-time translation tasks due to its rapidity (Maharjan et al., 2018). And the attention mechanism emphasizes that when facing a series of problems, it is more reasonable to allocate limited computing power, filter out high-value information, and improve processing efficiency (Han et al., 2020). It can get a relatively large accuracy improvement with a relatively small time consumption. Figure 9 shows the situation of each epoch during training.

**Figure 10:**
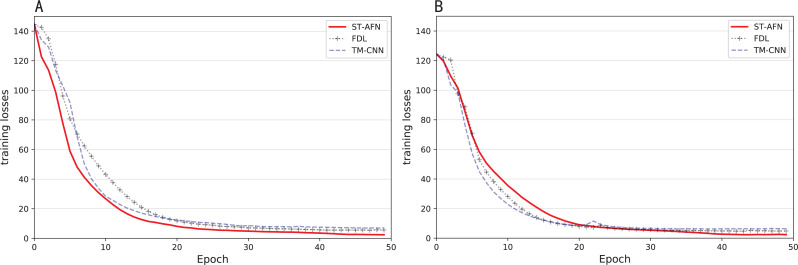
The situation of each epoch during training. (A) Training losses of Tonghui road. (B) Training losses of Shixin road.

It can be seen from Fig. 10 that three algorithms have completed training after 50 epochs. TM-CNN (in blue) improves quickly at the beginning, but its training is completed relatively early, and the final loss value is higher. ST-AFN’s training requires more epochs and achieves the best results. Figures 10A and 10B represent the results of Shixin Road and Tonghui Road respectively.

**Figure 11:**
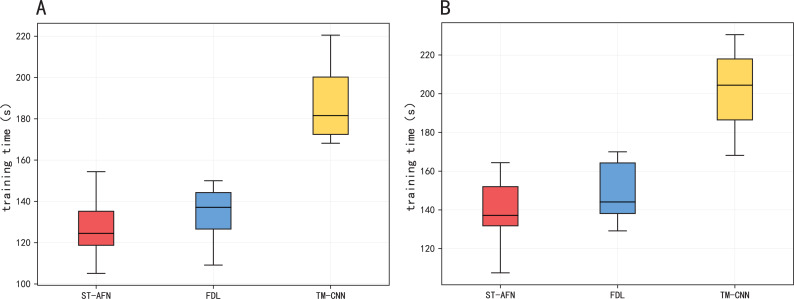
The training time of three models. (A) The training time of Tonghui road. (B) The training time of Shixin road.

Figure 11 and Table 6 show the training time of three models. Due to its seq2seq structure and attention module, ST-AFN training speed is slightly faster than FDL based on the gray correlation mechanism. TM-CNN takes longer because of its frequent convolution operation.

**Figure 12:**
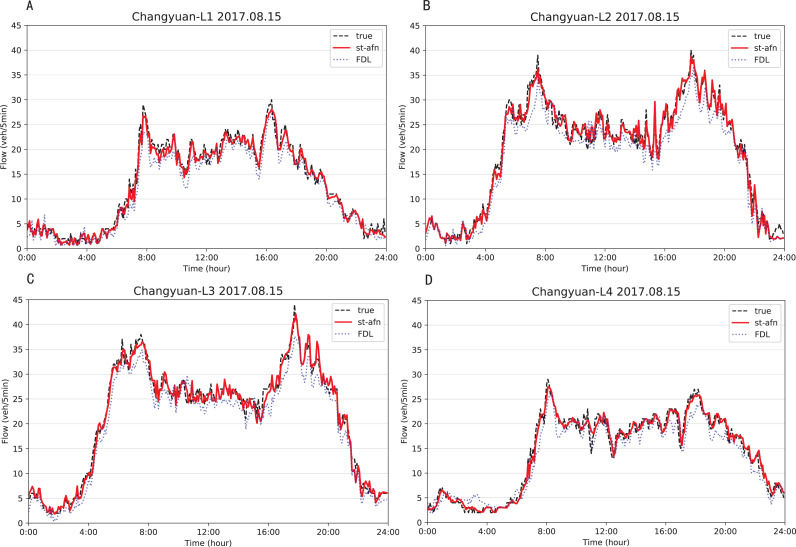
Comparison of ST-AFN with FDL on each lane (Tonghui Road). (A) The experimental result of Lane 1. (B) The experimental result of Lane 2. (C) The experimental result of Lane 3. (D) The experimental result of Lane 4.

**Figure 13:**
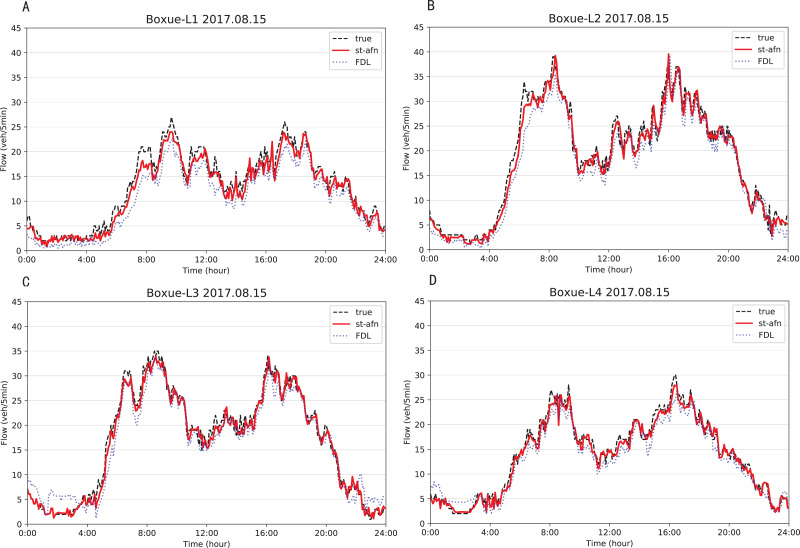
Comparison of ST-AFN with FDL on each lane (Shixin Road). (A) The experimental result of Lane 1. (B) The experimental result of Lane 2. (C) The experimental result of Lane 3. (D) The experimental result of Lane 4.

Figures 12, 13 show that ST-AFN outperforms the state of art model FDL on the prediction of traffic flow in each lane on a certain day. In the morning and evening peaks, this superiority is more obvious.

**Table 6:** Comparison among different models in training time.

**Algorithm**	Tonghui Road	Shixin Road
TM-CNN	190.54 s	204.37 s
FDL	133.99 s	145.72 s
ST-AFN	127.36 s	136.84 s

**Figure 14:**
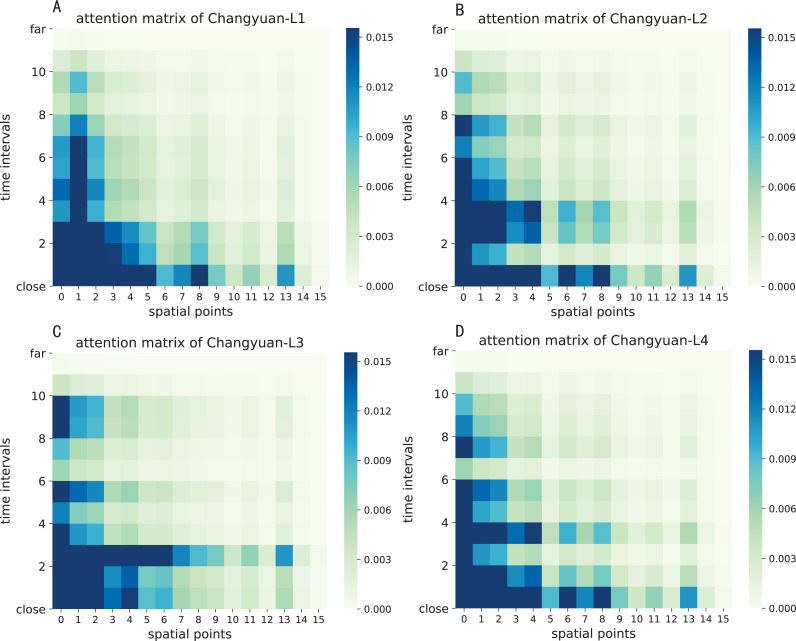
The attention result of ST-AFN (Tonghui Road). (A) The attention result of Lane 1. (B) The attention result of Lane 2. (C) The attention result of Lane 3. (D) The attention result of Lane 4.

**Figure 15:**
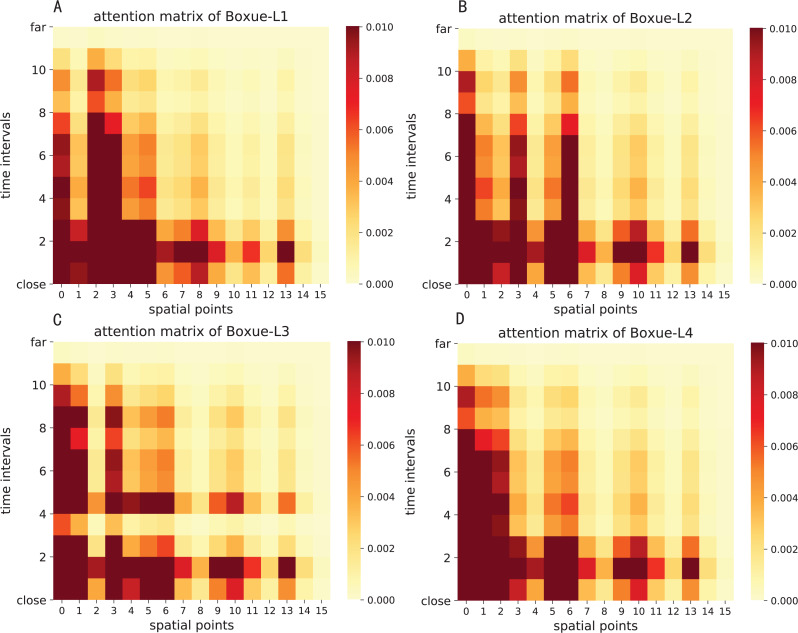
The attention result of ST-AFN (Shixin Road). (A) The attention result of Lane 1. (B) The attention result of Lane 2. (C) The attention result of Lane 3. (D) The attention result of Lane 4.

The attention result of ST-AFN is shown in the Figs. 14 and 15. In this picture, the *y*-axis represents the time intervals, and points on the *x*-axis represent the lanes. In detailed, we use the following lane sorting rules: Lanes closer to the target lane are assigned smaller values. If the distances are equal, it follows the order of upstream and downstream.

The darker the color of each point in the figure means that it achieves a greater weight score. From the result we can mainly summarize that the closer the time, the closer the distance, the greater the weight value will be. For the same distance, the upstream lane has a greater weight of influence compared to the downstream lane. Moreover, in this experiment, the temporal dependency plays a more important role than the spatial dependency.

## Conclusion

In this paper, we integrate attention mechanism with deep learning techniques to yield the spatial–temporal attention mechanism based fusion network (ST-AFN). Furthermore, a specific ground road lane selection method is also proposed to ST-AFN. The experimental results show that out model outperforms than the previous state of the art algorithms in lane-level traffic prediction.

## Additional Information and Declarations

### Data Availability

The following information was supplied regarding data availability: The data and code are available at GitHub: https://github.com/MCyutou/ST-AFN.
